# Evaluation of the feasibility, appropriateness, and acceptability of an environmental cleaning program improvement toolkit at a tertiary care hospital in Nigeria

**DOI:** 10.1186/s13756-025-01550-5

**Published:** 2025-04-18

**Authors:** Cori Dennison, Matthew Hudson, Damilola Adeniyi, Folasade Ogunsola, Hanako Osuka, Lisa P. Oakley, Paul Malpiedi, Amber Vasquez, Molly Patrick

**Affiliations:** 1 Division of Healthcare Quality Promotion, Centers for Disease Control and Prevention, Atlanta, GA USA; 2 Centre for Infection Control And Patient Safety (CICaPS), College of Medicine, University of Lagos, Lagos, Nigeria

**Keywords:** Healthcare environmental cleaning, Quality improvement, Resource-limited settings, Low- and middle-income countries

## Abstract

**Background:**

Environmental cleaning is a key infection prevention and control (IPC) intervention in healthcare settings. The U.S. Centers for Disease Control and Prevention (CDC), with Infection Control Africa Network (ICAN), developed best practices for global healthcare environmental cleaning in resource-limited settings to help fill gaps in guidance in low- and middle-income countries (LMICs). We aimed to evaluate the feasibility, appropriateness, and acceptability of a quality improvement toolkit developed to assist with implementing the CDC/ICAN best practices at Lagos University Teaching Hospital in Nigeria.

**Methods:**

A mixed-methods approach was used to evaluate the implementation of the toolkit from March through September of 2021. A monitoring checklist assessed feasibility after three defined steps within the toolkit. Key informant interviews and electronic surveys were conducted with toolkit team members at three time points during implementation to assess appropriateness and acceptability. A deductive analytic process was used to code and analyze interview data based on constructs of appropriateness and acceptability. Additional codes and sub-themes that emerged during analysis followed an inductive process.

**Results:**

Within the interviews and surveys, themes identified for the appropriateness included concern related to (1) time commitment for the toolkit activities and (2) resources required to sustain improvements. Themes identified for acceptability included (1) perceived challenges with time commitment and resource requirements, (2) perceived effectiveness of toolkit structure and usability, (3) perceived benefits and success associated with knowledge gained about environmental cleaning and environmental cleaning staff, (4) perceived benefits and success associated with the training for cleaning staff undertaken during toolkit implementation, and (5) perceived benefits and success associated with the multidisciplinary team approach with the inclusion of facility leadership and a project coordinator.

**Conclusions:**

The results showed that the toolkit materials were feasible within the local context and highlighted perceived effectiveness, benefits, and success of the toolkit process and experience contributing to a high level of acceptability. Challenges relating to time commitment and concern for sustainability have implications for the appropriateness of this toolkit, similar approaches to quality improvement, and the need for strengthening support for IPC improvements at the facility and national levels in resource-limited healthcare settings in LMICs.

**Supplementary Information:**

The online version contains supplementary material available at 10.1186/s13756-025-01550-5.

## Background

Healthcare-associated infections (HAI) are a significant burden globally and have been shown to disproportionately affect low- and middle-income countries (LMIC) [1]. While the World Health Organization’s (WHO) core components provide important building blocks for effective infection prevention and control (IPC) programs, notable gaps in facility implementation of the core components continue to hinder IPC progress [2]. A recent cross-sectional study by the WHO showed that only 15% of select facilities globally met all indicators considered as minimum requirements for IPC, with none of these being facilities in low-income countries [3]. Bolstering support for more effective and sustainable IPC programs is crucial to reducing pathogen transmission, HAI rates, and ensuring the safety of patients and healthcare workers.

An environment with heavy microbial contamination can play a role in pathogen transmission [4–6]. Therefore, environmental cleaning is recognized as a key IPC intervention. The effectiveness of environmental cleaning to reduce HAIs has been studied, but evidence remains limited regarding which specific environmental cleaning strategies or bundles most effectively reduce the risk of pathogen transmission [7–9]. While high-income countries have developed guidance using available evidence and best practices, guidance on environmental cleaning processes and programs that are feasible and effective in LMICs has been limited. In 2019, the U.S. Centers for Disease Control and Prevention (CDC), in collaboration with the Infection Control Africa Network (ICAN), published the *Best Practices for Environmental Cleaning in Healthcare Facilities in Resource-Limited Settings* to help fill this gap [10].

To support the progressive implementation of environmental cleaning programs as defined in the *Best Practices for Environmental Cleaning in Healthcare Facilities in Resource-Limited Settings*, CDC developed an Environmental Cleaning Program Implementation Toolkit. The specific objectives of the toolkit are to provide a standardized process for assessing environmental cleaning programs against the defined best practices, prioritizing needed actions and activities to make program improvements, and assisting in systematically implementing program improvements over time. It follows a stepwise, continuous quality improvement approach commonly used in IPC programs in both high- and limited-resource healthcare settings [11–13]. The toolkit follows a 5-step process for incremental program improvement (Table 1). Each step is undertaken by a facility-based, multidisciplinary project team, comprised of facility staff from varying departments of the hospital with differing roles including physicians, administrative staff, nurses, facilities management staff, IPC team members, and others as determined by the facility through the recommendations of the toolkit.

The toolkit was piloted in several settings to help validate and refine content prior to its publication in 2022 [14]. This formative evaluation describes the performance of the first pilot of the toolkit at Lagos University Teaching Hospital (LUTH) in Lagos, Nigeria from March through September of 2021, assessing the toolkit with respect to its feasibility, appropriateness, and acceptability.

## Methods

### Study setting

We conducted the evaluation at LUTH, a tertiary healthcare facility affiliated with the University of Lagos College of Medicine, which has 950 beds, 46 clinical departments, 18 non-clinical departments, and over 2,300 staff including medical laboratory scientists, medical officers, and nurses. A facility-level IPC program exists at LUTH, but staff are not assigned to work on IPC full-time. The LUTH IPC program began in the early 1990s and is supported by an IPC committee comprised of 33 members spanning 24 departments from across the hospital. The IPC team is comprised of 3 members and is responsible for implementing the policies of the IPC committee.

The environmental cleaning program at LUTH includes a mix of hospital-based environmental cleaning staff and outsourced environmental cleaning staff via contracts with several external companies. Prior to the toolkit implementation, there was not any hospital-level environmental cleaning policy, nor a standardized training program or training requirement for environmental cleaning staff.

As a part of the implementation of the environmental cleaning toolkit, LUTH established a multidisciplinary team to support the implementation of the toolkit activities. The implementation of the toolkit at LUTH was directed by a project coordinator and had a facility-based toolkit champion to lead activities. The team was comprised of 9 staff from 5 departments at LUTH. Once the multidisciplinary team was established, a risk assessment was conducted as a part of the toolkit implementation to determine hospital wards prioritized for program improvement [14]. Upon completion of the hospital-wide assessment, 19 high-risk patient care areas were highlighted out of which 2 special care neonatal units (Level II), one of 36 beds and one of 28 beds, were selected. These two neonatal units were selected based on the occurrence of recent HAI outbreaks and neonates being a vulnerable patient population group.

### Study design

A mixed-methods evaluation was designed to assess the feasibility, appropriateness, and acceptability of the toolkit among users. A structured monitoring checklist was used to assess the feasibility of toolkit activities at three defined steps within the 5-step process (after Step B, C and D). To assess appropriateness and acceptability, key-informant interviews and electronic surveys were conducted with members of the LUTH toolkit implementation team at three timepoints during the toolkit implementation process, after the Prepare for Action step in April 2021 (after Step A), after the Conduct Baseline Assessment step in May 2021 (after Step B), and after the Implement Program Improvements step in October 2021 (after Step D) (Table 1).

**Table 1:** Environmental cleaning program implementation toolkit 5-step approach to incremental program improvement and corresponding timepoints of data collection methods. All data collection occurred after the respective step it is listed under

	Implementation Toolkit Steps				
**Evaluation Method**	A. Prepare for action	B. Conduct baseline assessment	C. Develop action plan	D. Implement program improvements	E. Evaluate impact and sustain
Monitoring checklist		X	X	X	
Key informant interview	X	X		X	
Electronic Survey	X	X		X	

In this evaluation, feasibility was defined as the ability of the toolkit to achieve its intended purpose. Appropriateness was defined as whether the toolkit strategies were compatible with the operations and organization of the facility. Acceptability was defined as the perception of the toolkit by the participants and was comprised of several constructs adapted from a theoretical framework of acceptability previously designed by Sekhon and colleagues [15] (Table 2).

**Table 2:** Indicators, constructs, and sample key informant interview questions created to assess appropriateness and acceptability of toolkit implementation

Indicators/Constructs	Definition	Example Interview Guide Questions
1. Appropriateness	is the perceived fit and compatibility of the toolkit relative to the current organization and operation of the facility	• Did you notice that there were any things about the toolkit that didn’t apply or didn’t really work at your facility? • What changes could have been made to this section of the Toolkit (or previous parts) to better meet the needs at the facility?
2. Acceptability	refers to the emotional and cognitive perceptions of the toolkit by the participants and is further defined by the constructs below	*See constructs listed below*
*Affective attitude*	refers to users’ feelings about applying/implementing the toolkit	• How would you describe your ability to identify areas for improvement in environmental cleaning at your facility? • How did you find your role as […] during this Section of the toolkit?
*Burden*	refers to the amount of effort required to complete the toolkit	• Did parts of this Section require too much effort or time from the team members?
*Experience*	refers to the successes and challenges encountered while using the toolkit	• Which tools of this Section worked well? Was there anything that was confusing?
*Intention*	refers to the willingness of the user to participate in the toolkit process	• What was your role during this part of the Toolkit?
*Opportunity costs*	refers to time and resources perceived as lost by the facility because of using the toolkit	• Did the time dedicated to the Toolkit cause you to not be able to complete your regular work duties?
*Perceived effectiveness*	refers to the extent to which the intervention is perceived as likely to achieve its purpose	• What would you say this part of the Toolkit accomplished?

### Roles and responsibilities

The CDC evaluation team worked closely with the project coordinator at LUTH to provide supervision and technical support to the implementation of toolkit activities and evaluation at the three timepoints of implementation. LUTH provided ethical review and approvals for the implementation and evaluation of the toolkit. The LUTH project team, including the project coordinator, implemented the toolkit activities at the facility. Team members participated in the evaluation of the toolkit throughout its implementation.

### Data collection

Structured monitoring checklists (Additional file 1) were used to assess feasibility of the toolkit activities and associated tools for each of the required steps. The checklists were completed by the CDC evaluation team based on document review of the toolkit documents, such as the baseline assessment, to assess to what degree the toolkit steps were followed and completed.

Key informant interviews were arranged by the project coordinator and conducted by the CDC evaluation team with a selected group of eight members of the LUTH project team. Questions in the interview guide were informed by the appropriateness and acceptability indicators and their associated constructs and were complemented in real-time by additional prompts and responses provided (Additional file 2). Interviews were conducted, recorded, and transcribed using Zoom technology (Zoom Video Communications, Inc.; San Jose, California) with evaluation team members reviewing transcriptions.

Electronic surveys (Additional file 3) were administered using Survey Monkey (Momentive; San Mateo, CA) and supplemented data on appropriateness and acceptability by measuring level of agreement with Likert-type statements. These surveys were completed by the same toolkit staff participating in the key informant interviews and at the same time points as the interviews.

### Data analysis

A deductive analytic process was used to code and analyze interview data based on constructs of appropriateness and acceptability. Additional codes and sub-themes that emerged during analysis followed an inductive process.

The evaluation team used MAXQDA Plus 2022 (VERBI Software; Berlin Germany) to analyze and code responses. For quality assurance, three transcripts were initially coded by three different members of the CDC evaluation team and compared as a group to ensure compatibility of coding between individuals and consensus for introducing and defining the relevant sub-themes that emerged towards the evaluation objectives. Following this, the remainder of transcripts were divided and coded by one of three individual evaluation team members. Following coding of the transcripts, initial thematic analysis was conducted for each code by two members of the CDC evaluation team, followed by a combined thematic analysis to general overall themes and sub-themes. Data were additionally stratified by respondent type (e.g., physician, nurse, etc.) and timepoint of the interview to determine any specific themes based on those strata. Themes and sub-themes were then categorized under the appropriateness and acceptability indicators.

Survey data were analyzed by importing anonymous responses from Survey Monkey into Excel (Microsoft; Redmond, WA). Data were analyzed by each timepoint of the toolkit implementation.

### Ethical considerations

The evaluation protocol received was reviewed and approved by the LUTH Health Research Ethics Committee. This activity was reviewed by CDC and was consistent with applicable federal law and CDC policy (*See e.g.*,* 45 C.F.R. part 46*,* 21 C.F.R. part 56; 42 U.S.C. § 241(d); 5 U.S.C. § 552a; 44 U.S.C. § 3501 et seq.*). Participation in all interviews was voluntary and informed. All participants provided verbal consent prior to recording of interviews via teleconference using Zoom. Electronic surveys did not collect any personally identifiable information and transcripts from key informant interviews were de-identified prior to analysis.

## Results

The toolkit team was comprised of eight members– four physicians, two nurses, one administrator, and one engineer/facilities management staff member. At least five of the team members participated in the evaluation interviews and cross-sectional surveys across each time point of data collection.

### Feasibility

Data collected using the monitoring checklist indicated completion of every step and activity outlined. The document review of the checklists resulted in all steps of the toolkit implementation being completed by the team with the tools provided.

### Appropriateness

Two key themes of appropriateness of the toolkit, time commitment and resources required, were identified through key informant interviews.

### Time commitment required for the toolkit by team members

When discussing the perceived fit and compatibility of the toolkit, team members expressed that dedicated time was not given to them to perform their assigned activities for the project in addition to their regular duties. Team members described that they were often asked to perform the toolkit activities alongside their regular facility duties making it difficult to commit time to the toolkit.

*“…the way we work*,* I think our system*,* we do not allocate special time for different activities. So*,* you are a microbiologist*,* you are in infection control*,* you are in antibiotic stewardship*,* you teach*,* you train. You just do everything the way that you [should] when it comes up. So*,* there’s no dedicated time to say you need to do two hours of antimicrobial stewardship in a week or do six hours of this in the day. So*,* everything is all lumped together. You need to find a balance with all these activities” - Physician 1*.

**Fig. 1:**
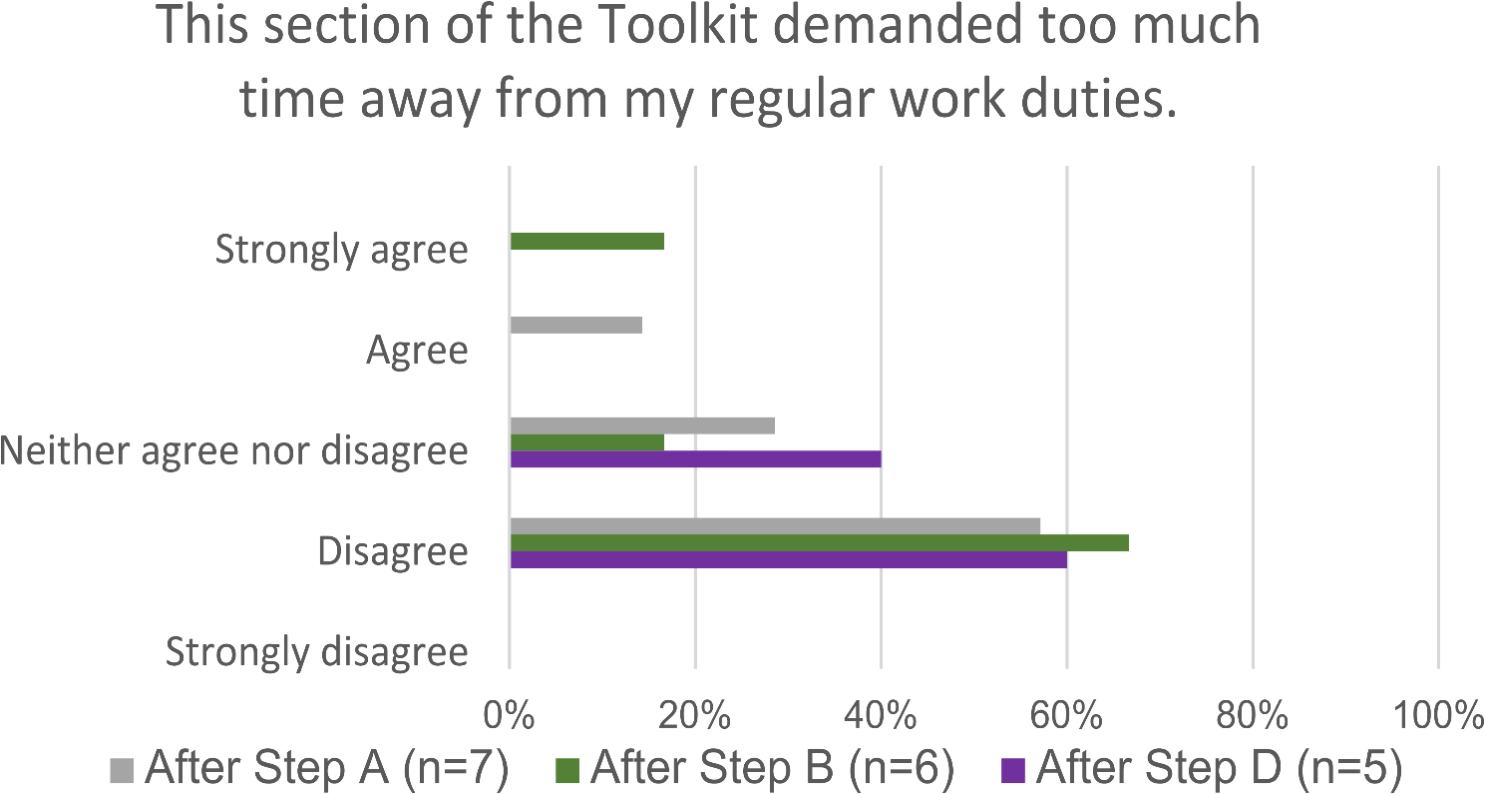
Electronic survey data analysis comparing participant answers to the perceived time commitment (Burden) for the toolkit after Steps A, B & D of the toolkit implementation at LUTH in Lagos, Nigeria, 2021. (n = number of participants)

Survey results relating to time commitment varied throughout at the different time points of data collection. While most participants stated that the toolkit did not demand too much time away from their work duties, others disagreed, specifically after Step (A) Prepare for Action and Step (B) Conduct baseline assessment (Fig. 1). However, despite facing challenges in the lack of dedicated time provided to team members, time spent on the toolkit was seen as time well spent and worthwhile for the team.

*“…but the meetings doesn’t [sic] take so much time and some of them are a mixture of virtual meetings and then physical meeting so it didn’t take*,* really if we consider it*,* is not too much and it has been a fruitful time for all of us.”– Physician 2*.

### Resources required to sustain toolkit activities

Team members expressed concerns that resources may not be available within the facility to support lasting changes identified by the toolkit. Team members described that a lack of funding for environmental cleaning may limit the sustainability of improvements in the future at the facility and that leadership allocation of funds would be necessary for continued improvement after implementation.

*“We need financial assistance. We need funds. You know*,* to get the materials*,* to get equipment*,* we need funds. We need management to inject funds. Because when you put everything together*,* if there is no backup fund then at the end of the day*,* we have not done anything that can last. So*,* we need management to inject funds for the project.”– Engineer*.

Team members also cited concerns with a lack of appropriate materials (e.g., disinfectants) and equipment (e.g., mops) to support the implementation of cleaning procedures according to the best practices which were taught during the training, rolled out to frontline cleaning staff during the project, and included in new standard operating procedures developed by the project team.

### Acceptability

Five key themes were identified in key informant interviews relating to constructs of acceptability: one of which described perceived challenges and overlapped with themes identified as relating to appropriateness and four distinct themes which generally described perceived effectiveness and successes associated with the toolkit.

### Perceptions of time commitment and resource requirements to sustain toolkit activities

While the issues of time commitment and resources were described within the context of the facility operations and structure, these themes also influenced acceptability as the toolkit members perceived them as challenges within their experience of the toolkit. Lack of dedicated time given for toolkit activities was described as a function of the facility; however, time management was also perceived as a general challenge throughout the toolkit process. Perceptions about time management were discussed mostly during interviews after Step A. Prepare for action). Team meetings were described as a challenge among team members due to conflicting work schedules. However, this was compensated for by arranging short retreats on the weekend and a mix of in-person and virtual meetings for toolkit related activities.

*“Getting together unified [sic] to activate the group for this purpose can be difficult*,* because everyone has his own schedule*,* so that [is] the only challenge I would say that I’m experiencing presently.”– Engineer*.

Team members also perceived challenges in the availability of materials to sustain improvements made. Most comments related to these challenges were brought up during interviews conducted after Step D. Implement program improvements.

*“…another area of challenges is the materials itself*,* because in as much as there are [sic] dedicated staff or a person that is willing to do work*,* if you’re not provided with everything necessary that you need to carry out the work*,* then it brings the difficulty to the table.”– Nurse 1*.

### Perceptions of toolkit structure and usability of toolkit materials

The design of the toolkit and its accompanying implementation tools were perceived to be effective at identifying gaps and enabling improvements to be made on environmental cleaning best practices. The toolkit was described as simple and easy to use by team members. The simplicity of the toolkit, its design, and materials made it flexible for facility needs, allowing for ease of adaptability.

“…*you cannot take away the role of having a systemic process in place. So*,* with this toolkit there is already a structure in place. So*,* it made it easy–we probably would have stumbled our way and spend [sic] more time planning*,* so the toolkit saves time and pointed us in the right direction at the right time*,* so there’s a lot of time saved. And*,* also*,* the process—we’ve learned how to go approach such problems now with a process*,* a stepwise process*,* so it took it was very helpful.”– Physician 3*.

Most team members expressed that some adaptation of the toolkit tools (e.g., baseline assessment questions) were required. Once adapted to the context of the facility or ward, the tools were perceived as useful.

*“It [toolkit implementation tool] was not perfect for my environment*,* but I used it in line with what’s available to our procurement departments. So*,* I was able to visit the procurements section*,* and then see what was available. So based on the suggestion or sample of what I have in the toolkit*,* I used it to form and develop what I could present and that was brought up in a meeting and then to defend it*,* to explain it*,* and then the modifications was [sic] done before the final submission.” - Nurse 1*.

### Perceptions and knowledge about environmental cleaning and environmental cleaning staff

Toolkit team members expressed that the toolkit was successful in increasing their knowledge and awareness for environmental cleaning practices in their healthcare facility. The toolkit provided a new and organized way to learn about environmental cleaning, which changed team members’ perceptions and increased their appreciation for the importance of environmental cleaning and environmental cleaning staff for IPC.

*“My experience with working on the team has been an eye opener for me*,* even as the maintenance officer… We need to make the environment more conducive for the cleaners and the patients. The way they were disposing water before was inappropriate. We need a separate way to remove sluice water than the multiuse sinks.” - Engineer*.

Particularly among the physicians, they reported positive perceptions related to their increased knowledge, citing understanding how to prepare disinfectants, how frequently cleaning should take place on the units, and the cleaning procedures themselves. They also expressed an appreciation for the fact that environmental cleaning staff are part of the IPC team.

*“This has emphasized the role of teamwork in whatever we want to do. If we want to prevent infection in the neonatal unit*,* we cannot overlook some people. Everybody has to work hand in hand and the cleaners are part of that team. And we start with them*,* because if they don’t understand what it is that they are doing and why they are doing*,* they will be on their own. Everybody has to be carried along and the cleaners and team have to understand why it’s important that we prevent infections in our newborn*,* so I think this has really opened my eyes and also taught me that I shouldn’t be neglecting some segments that are part of it. They have to be involved.”– Physician 4*.

**Fig. 2:**
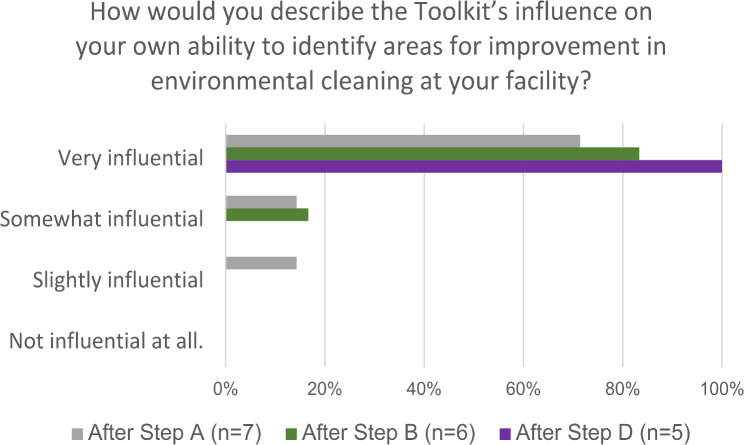
Electronic survey data analysis comparing participant answers to the perceived ability to identify areas of improvement (Affective Attitude) after Steps **A**, **B** & D of the toolkit implementation at LUTH in Lagos, Nigeria, 2021 (n = number of participants)

Within the survey results, team members indicated that the toolkit was influential in their abilities to identify areas of improvement in environmental cleaning in some capacity across all time points. The perceived increased ability to identify areas of improvement was found to be most influential after Step D. Implement program improvements, after they’d both identified and implemented improvement activities (Fig. 2).

### Perceptions about training for cleaning staff undertaken as part of the toolkit implementation

The creation and implementation of a training for facility cleaning staff designed by the LUTH toolkit team was perceived as beneficial and successful. The training, which included cleaning staff, clinical staff from the neonatal units, and facility leadership, was described as helpful in bringing to light the importance of training and engaging cleaners in the environmental cleaning process at the facility.

*“[The training] was effective in that people have acquired basic knowledge. A lot of times we tend to think that all we’ve been doing cleaning*,* we know [sic]. But in identifying these gaps we now know better*,* how to clean*,* where to target*,* how to clean from where to where*,* even the storage of all the cleaning materials and all that*. *So it was effective*,* a lot of knowledge was impacted.”– Physician 3*.

Toolkit team members also expressed seeing the impact of the training in terms of the practices of cleaning staff as well as their motivation on the wards.

“…*cleaners’ attitudes are now changed*,* cleaners’ motivation and*,* yes*,* and that is [sic]*,* served as a source of change. We now have more cleaners coming in to say*,* I want to be part of it and get it right so*,* we achieved that. And if these things as efficient [sic] as it is expected*,* I think we have it better.”– Nurse 1*.

### Perceptions about the multidisciplinary team approach with the inclusion of facility leadership and the involvement of a project coordinator

Members of the toolkit team recounted the benefits of working in a multidisciplinary team as a part of toolkit implementation. The opportunity to work with staff across departments at the facility was perceived as contributing to the success of the toolkit implementation. Involvement of facility leadership (e.g., Chairman Medical Advisory Committee (CMAC)) in the toolkit process was perceived to improve impact and the sustainability of improvements.

*“I’m also glad that this project is coming up with involvement with the top managerial*,* like the CMAC is there*,* so it’s easier when you want to inculcate things like this into the system*…*because if he is not there to follow to all the activities*,* it might just be you pouring water on stone by the time you’re done. It’s just there. So*,* I also like the fact that the top people who are responsible*,* they are there.*” - *Nurse 2*.

The importance of the project coordinator also attributed to the perceived success of the implementation of the toolkit. Support from the project coordinator alleviated challenges with implementation by providing support and structure to the team throughout the implementation process. The perceived importance of the project coordinator to the success of the toolkit implementation was mentioned largely by the nurses in the toolkit team during Time Point 2, at a critical time when decisions on improvement activities to prioritize had to take place.

*If he [project coordinator] wasn’t there*,* it will [sic] have been difficult to use.”– Nurse 2*.

The team members also cited the project coordinator’s role in preparing documents and agendas in advance of team meetings and facilitating meetings to keep team members focused.

## Discussion

The evaluation results indicate that completion of the toolkit activities was feasible throughout the implementation process at LUTH. The monitoring checklists at each time point indicated that every step of the process and associated materials (e.g., baseline assessment) were able to be completed according to their purpose and with the tools provided in the toolkit. Interviews highlighted that some of the tools had to be adapted to the context of the facility, but the perceived flexible design, simplicity, and ease of use of the tools enabled these adaptations. Team members indicated in both the cross-sectional survey and interviews that the toolkit provided increased knowledge and was effective at enabling improvements to environmental cleaning, which provides further evidence supporting the overall feasibility of the process and tools.

Despite the toolkit and associated tools being described by team members as effective and easy to use, there were reported challenges with implementing the toolkit at LUTH due to lack of dedicated time for toolkit activities and associated difficulties in coordinating already busy schedules. Relatedly, team members frequently mentioned that the support of the project coordinator was essential to overcome some of these challenges, given that the coordinator had dedicated time for organizing meetings and keeping track of deadlines for activity implementation. The challenges relating to high workload and lack of dedicated time for quality improvement have also been highlighted in a quality improvement collaborative initiative in Ethiopia, where the ability of individual facilities to continue with quality improvement projects was limited by a lack of staff time and, similarly, was perceived to require support from external mentors from non-governmental organizations [16]. In this evaluation, interviewees discussed that, beyond the scope of the toolkit project, many of them assume multiple roles within the facility due to understaffing. A lack of adequate healthcare workforce in LMICs have been shown to have negative impacts at the individual and healthcare system level [17].

At the end of the toolkit implementation there was an expressed concern in the interviews about the lack of resources available (e.g., supplies and equipment) at the facility to sustain the improvements and the need for leadership to make resources available for sustainability. A recent systematic review by Zamboni et al. highlights the need for sufficient facility organizational structures, such as budget, as important factors for sustaining outcomes of quality improvement collaboratives [18]. The importance of adequate budget has also been documented in a study from 2017 by Fejfar et al. where results from the analysis of healthcare provider satisfaction with environmental conditions in fourteen LMICs in Africa discovered a significant association between aspects of institutional support, including sufficient budgeting, and increased satisfaction of cleanliness and IPC practices [19].

This evaluation highlighted several positive perceptions regarding the overall experience of implementing the toolkit. The multidisciplinary team approach recommended in the toolkit, which included leadership, was perceived positively. Increased knowledge and awareness of environmental cleaning and inclusion of environmental cleaning staff as key members of the IPC team was also reported as a positive. The benefits of multidisciplinary teams and leadership involvement, as part of a multimodal strategy, have been demonstrated in IPC improvement initiatives in other LMICs. A study in Colombia demonstrated that inclusion of multidisciplinary teams during project implementation improved patient care systems, and a study in Vietnam using all elements of the WHO multimodal strategy, including leadership involvement, showed contribution to positive outcomes in a long-term hand hygiene improvement initiative (20–21). The importance of leadership involvement and buy-in in IPC implementation efforts has also been recently highlighted by Tomczyk et al. within a qualitative analysis identifying themes associated with effective implementation of the eight core components of IPC in low-resource settings [22]. In this study, the importance of leadership buy-in to effectively implement multimodal strategies is cited, as well as the need to regularly engage leadership to improve IPC programs at the facility level.

Notably, as a part of the action plan during toolkit implementation, the LUTH facility team decided to develop and implement a training program for their facility cleaning staff. The implementation of this large training program was outside of the scope of the toolkit itself; however, it was a major contributor in the thematic analysis to the increase in knowledge and awareness of the importance of environmental cleaning and was perceived as beneficial and successful in the context of the project. This finding supports the benefits of implementing quality improvement collaboratives that include a training component as found in previous studies [23]. Storr et al. highlight that training of cleaning staff has the potential to provide impactful relationships with other healthcare workers at a facility and create an atmosphere where environmental cleaning staff are recognized as being valued members of a workforce [24]. Often, environmental cleaners are not prioritized by the facility in terms of support and training and are seen as an ‘invisible workforce’. A study published in 2019 demonstrated that a lack of understanding among clinical staff on cleaners’ jobs and knowledge of their roles has been shown to negatively affect quality of training and contribute to poor environmental cleaning practices and ultimately low job satisfaction by cleaners [25]. Results from our evaluation support the benefit of involving nurses, physicians, and leadership in environmental cleaning training alongside environmental cleaning staff, as it contributed to increased awareness and understanding for the importance of cleaners and the role they play in the facility workforce.

There are several limitations with this evaluation. First, while the evaluation was primarily qualitative, the number of team members participating in interviews was small, and we did not include feedback from the broader set of participants who were peripherally impacted by the toolkit process, such as the environmental cleaning staff or other clinical staff on the priority wards. Second, while not intended at the start of this study, the hired project coordinator took on a central leadership role in implementing the toolkit at LUTH, which may have affected the evaluation results related to the perceived success. Similar success may not have been seen without the central leadership role of the project coordinator. Third, the platform in which the interviews were given, Zoom, may have yielded different interview results than if they had been administered in person. For example, connection issues periodically disrupted verbal communication, nonverbal communication was also more limited than it would have been during an in-person interview, and the virtual platform did not allow for the CDC team to decide on a location for both participants and interviewers that was free from potential distractions that could have influenced data collection and results. Lastly, the evaluation of the toolkit implementation was led by U.S. CDC, which may have led to social desirability bias among respondents.

## Conclusion

This evaluation sought to determine the feasibility, appropriateness, and acceptability of the CDC/ICAN Environmental Cleaning Program Implementation Toolkit in a resource-limited setting. While the toolkit process and materials were feasible among the toolkit team members, challenges with time commitment and concern for sustainability presented themselves throughout the evaluation. These challenges may have implications for the level of appropriateness of an in-depth quality improvement process in the context of a resource-limited setting without external support (e.g., a project coordinator), and speak to the broader need to strengthen support for IPC improvements at both facility and national levels in LMICs. Perceived effectiveness, successes, and benefits associated with the toolkit process and overall experience contributed to the high level of acceptability. Importantly, this evaluation supports the benefits of involving and elevating environmental cleaning staff as a part of environmental cleaning improvement activities in LMICs and involving leadership as a part of a multidisciplinary IPC improvement team. Finally, while these results help validate an implementation process for the CDC/ICAN Best Practices for Environmental Cleaning, more research is needed to define and prioritize the elements of a multimodal strategy for environmental cleaning that leads to sustained and effective cleaning practices in resource-limited settings.

## Electronic supplementary material

Below is the link to the electronic supplementary material.

Supplementary Material 1

Supplementary Material 2

Supplementary Material 3

## Data Availability

No datasets were generated or analysed during the current study.

## Abbreviations

HAI: Healthcare associated infection
LMIC: Low- and middle– income country
WHO: World Health Organization
IPC: Infection prevention and control
CDC: Centers for Disease Control and Prevention
ICAN: Infection Control Africa Network
LUTH: Lagos University Teaching Hospital
