# Supplementary material for: Evaluation of the feasibility, appropriateness, and acceptability of an environmental cleaning program improvement toolkit at a tertiary care hospital in Nigeria
Source: Antimicrob Resist Infect Control. 2025 Apr 18;14:33. doi: 10.1186/s13756-025-01550-5 (PMC12008941; doi:10.1186/s13756-025-01550-5)
Supplement: Supplementary file 1 — Supplementary Material 1 [file 13756_2025_1550_MOESM1_ESM.docx]

**Additional file 1: Monitoring Checklist**

| **Prepare for Action Checklist (Performed after completion of Section A)** | | |  |  |  |  |
| --- | --- | --- | --- | --- | --- | --- |
| **Preparer:** This is to be completed by an evaluation team designee | | | |  |  |  |
| **Timing:** This checklist should be completed soon after completion of the final step of Section A (Prepare for Action) | | | |  |  |  |
| **Instructions**: This data will be collected by the preparer through a review of Associated Documents noted below and reflect the fulfillment of the intended steps within the Section. | | | |  |  |  |
| **Associated Documents:** Team roster (TBD), Terms of Reference, Risk Classification of Functional Areas | | | |  |  |  |
| **Questions** | **No=0, Yes=1** | **Comment** | | | | |
| **The following team members were successfully identified:** *(If NO, please note which entries are missing responses and associated comments)* |  | | | | | |
| *Team leader* |  |  | | | | |
| *IPC representative* |  |  | | | | |
| *Nursing department representative* |  |  | | | | |
| *Facilities management representative* |  |  | | | | |
| *Administrative representative* |  |  | | | | |
| *Priority ward representative* |  |  | | | | |
| **Were terms of reference created for all roles?** *(If NO, please comment regarding which ToRs were not completed)* |  |  | | | | |
| **Was a risk classification of functional areas performed?** |  |  | | | | |
| **If YES, which wards were included? (Text)** |  | | | | | |
| **Were risk scores calculated for the wards classified?** *(If only some included wards were classified, answer "SOME" and please note incomplete wards)* |  | | | | | |
| **Total score** |  |  | | | | |
| **Risk status** |  |  | | | | |
| **Were the following fields for the Risk Classification of Functional Areas table filled in correctly based on toolkit instructions?** *(Did sums add up and/or correlate with risk assessed)* |  | | | | | |
| **Total score** |  |  | | | | |
| **Risk status** |  |  | | | | |
| **Was a priority ward selected for further assessment and action? (If NO, please comment on cited reasons)** |  |  | | | | |
| **What factors contributed to the ward’s selection?** (If applicable) |  | | | | | |
| *Risk classification score(s)* |  |  | | | | |
| *Recent HAI outbreaks* |  |  | | | | |
| *Rates of HAIs from ongoing surveillance* |  |  | | | | |
| *Facility-supported QI initiatives (e.g., hand hygiene)* |  |  | | | | |
| *Priority patient populations* |  |  | | | | |
| *Government-supported QI initiatives* |  |  | | | | |
| *Internal funding for QI initiatives* |  |  | | | | |
| *External funding for QI initiatives (e.g., from government)* |  |  | | | | |
| *Other (Please comment)* |  |  | | | | |
| **Additional notes/comments:** |  |  | | | | |

| **Baseline Assessment Checklist (Performed after completion of Section B)** | |  |  |  |  |
| --- | --- | --- | --- | --- | --- |
| **Preparer:** This is to be completed by an evaluation team designee | |  |  |  |  |
| **Timing:** This checklist should be completed after completion of Section B (Baseline Assessment) | |  |  |  |  |
| **Instructions:** This data will be collected by the preparer through a review of Associated Documents noted below and reflect the fulfillment of the intended steps within the Section. | |  |  |  |  |
| **Associated Documents:** Baseline Program Assessment, Leadership Interview, Cleaning Staff Interview, Ward & Services Assessment | |  |  |  |  |
| **Questions** | **No=0, Yes=1** | **Comment** | | | |
| **Was Part 1 - Basic Program Assessment completed?** |  |  | | | |
| **Were all of the indicators within each of the five program elements assessed in Part 1?** *(If NO, please note which indicators are missing for each element and reasons provided for absence)* |  |  | | | |
| *Organizational elements* |  |  | | | |
| *Policy and procedural elements* |  |  | | | |
| *Staffing and training elements* |  |  | | | |
| *Infrastructure and supply elements* |  |  | | | |
| *Monitoring and feedback elements* |  |  | | | |
| **Was Part 2 – Advanced program assessment completed?** | 0 |  | | | |
| **Were all of the indicators within each of the five program elements assessed in Part 2?** *(If NO, please note which indicators are missing for each element and reasons provided for absence)* | 0 |  | | | |
| *Organizational elements* |  |  | | | |
| *Policy and procedural elements* |  |  | | | |
| *Staffing and training elements* |  |  | | | |
| *Infrastructure and supply elements* |  |  | | | |
| *Monitoring and feedback elements* |  |  | | | |
| **Was a leadership interview performed?** |  |  | | | |
| *Were all entries complete? (If NOT, please note which entries are missing responses and associated comments)* |  |  | | | |
| *Were the following documents provided to the project team for review?* |  |  | | | |
| *1. Facility cleaning policy* |  |  | | | |
| *2. Service Agreement or Contract with cleaning service company* |  |  | | | |
| *3. Job descriptions, terms of reference for:* |  |  | | | |
| *a. Cleaning Focal Point* |  |  | | | |
| *b. Cleaning Supervisors* |  |  | | | |
| *c. Cleaning Staff* |  |  | | | |
| *4. Cleaning supplies & equipment list from procurement department* |  |  | | | |
| *5. Cleaning program budget* |  |  | | | |
| *6. Organizational staffing chart for facility or priority ward* |  |  | | | |
| *7. Cleaning staff roster* |  |  | | | |
| **Was a cleaning staff interview performed?** |  |  | | | |
| *Were all entries complete? (If NOT, please note which entries are  missing responses and associated comments)* |  |  | | | |
| **Was a ward and services assessment performed?** |  |  | | | |
| **Were all entries complete for the following areas of the assessment? (If NO, were explanations provided for incomplete responses)** |  |  | | | |
| *1. Staffing* |  |  | | | |
| *2. Policies and procedures* |  |  | | | |
| *3. Supplies and equipment* |  |  | | | |
| *4. Environmental services storage area* |  |  | | | |
| *5. Sluice area* |  |  | | | |

| **Recommended Action Checklist (Performed after completion of Section C)** | |  |  |  |  |
| --- | --- | --- | --- | --- | --- |
| **Preparer:** This is to be completed by an evaluation team designee | |  |  |  |  |
| **Timing:** This checklist should be completed after completion of Section C (Recommended Action) | |  |  |  |  |
| **Instructions:** This data will be collected by the preparer through a review of Associated Documents noted below and reflect the fulfillment of the intended steps within the Section. | |  |  |  |  |
| **Associated Documents:** Baseline Assessment Key, Recommended Actions Guide, Action Plan Template, | |  |  |  |  |
| **Questions** | **No=0, Yes=1** | **Comment** | | | |
| **Was a Baseline Assessment Key produced/performed?** |  |  | | | |
| *Were all elements present and all fields are completed? (If NO, were explanations provided for incomplete responses)* |  |  | | | |
| *Did priority groups match those of the Recommended Actions Guide?* |  |  | | | |
| *How many deficits were flagged for an action plan?* |  |  | | | |
| *From which priority group(s) were these deficits flagged? (Please list)* |  | | | | |
| **Are all element codes for intervention listed in the Action Plan Template?** |  |  | | | |
| **Do action(s) to take correspond with element codes per Recommended Actions Guide?** |  |  | | | |
| **Are actions/activities listed that are not included in the Recommended Actions Guide? (If YES, please elaborate)** |  |  | | | |
| **Do each of the listed actions in the Action Plan have the following elements filled in? (If NO, please note which entries are missing responses and associated comments)** |  | | | | |
| *Action(s) to take* |  |  | | | |
| *Persons responsible* |  |  | | | |
| *Resources needed* |  |  | | | |
| *Target due date* | 1 |  | | | |
| **Was a Gantt chart created for Action Plan activities?** |  |  | | | |

| **Implementation & Evaluation Checklist (Performed after completion of Section D)** | | |  |  |  |  |
| --- | --- | --- | --- | --- | --- | --- |
| **Preparer:** This is to be completed by an evaluation team designee | | | |  |  |  |
| **Timing:** This checklist should be completed between 6-8 months after initiation of the toolkit. | | | |  |  |  |
| **Instructions:** This data will be collected by the preparer through a review of Associated Documents noted below and reflect the fulfillment of the intended steps within the Section. | | | |  |  |  |
| **Associated Documents:** Action Plan, Final Report | | | |  |  |  |
| **Questions** | **No=0, Yes=1** | **Comment** | | | | |
| **Were any Program Improvement tools used for the program improvement activities undertaken?** |  |  | | | | |
| **If YES, which Program Improvement tools were used for the program improvement activities undertaken? (Please list)** |  | | | | | |
| **Have there been updates/edits to the original Action Plan?** *(If YES, please comment on completeness of the additions and any underlying factors contributing to changes)* |  |  | | | | |
| **Have all Action Plan activities been implemented?** |  |  | | | | |
| **If not, were reasons provided for activities not being implemented?** |  |  | | | | |
| **Were all of the listed actions/activities in the Action Plan completed?** |  |  | | | | |
| **If no, which actions were not completed** |  |  | | | | |
| **Were all planned activities completed by the target date noted on the Action Plan? (If NO, what reasons were provided for delays?)** |  |  | | | | |
| **Was a final report created?** |  |  | | | | |
| **Was the final report template used?** |  |  | | | | |
| **If YES, were the sections complete? (If NO, note explanations or comments offered for the incomplete document)** |  |  | | | | |
| **Were there components included in the final report that were not in the provided template?** |  |  | | | | |
| **Have monitoring activities been described to support the Action Plan activities?** |  |  | | | | |
| **If NOT, were reasons provided for excluding monitoring activities from the plan?** |  |  | | | | |
| **Is a monitoring audit clearly defined?** |  |  | | | | |
| **Is there a plan to revisit the toolkit process?** |  |  | | | | |
| *Is the timeline currently defined?* |  |  | | | | |
