# Supplementary material for: Evaluation of the feasibility, appropriateness, and acceptability of an environmental cleaning program improvement toolkit at a tertiary care hospital in Nigeria
Source: Antimicrob Resist Infect Control. 2025 Apr 18;14:33. doi: 10.1186/s13756-025-01550-5 (PMC12008941; doi:10.1186/s13756-025-01550-5)
Supplement: Supplementary file 2 — Supplementary Material 2 [file 13756_2025_1550_MOESM2_ESM.docx]

**Additional file 2. Key Informant Interview Guide**

**Assessment of the applicability and effectiveness of the Environmental Cleaning Toolkit implementation at LUTH in Lagos, Nigeria: Interview Guide for the [Insert Section]**

**Date:**

**Interviewer Name:**

**Notetaker Name:**

**Participant ID:**

**Occupation:**

*(Instructions to notetaker: Participant ID: notate as interviewer’s initials_MMDD_interview number (ex. VF_0823_01)). The notetaker will manage the recording and will start recording at the end of the verbal consent (notated below). Please refer to note taker document for further guidance)*

Good morning/afternoon. Thank you for taking the time to speak with us today about your experiences with the implementation of the Environmental Cleaning Toolkit at LUTH. I am [NAME OF INTERVIEWER] and I am joined by [NAME] who will be taking notes during our discussion. This part of a voluntary project is being conducted by the Centers for Disease Control and Prevention. The goal of this project is to evaluate how feasible, acceptable, and appropriate the toolkit is for helping hospitals to make improvements to their environmental cleaning programs. The information we collect during this interview will be used to make adjustments and additions to the toolkit so that it is more effective and usable. We very much appreciate you making time to provide your inputs towards this goal.

These interviews throughout the course of the implementation of the environmental cleaning toolkit are being conducted to inform the specific improvements to each section of the toolkit - Prepare for Action, Baseline Assessment, Recommended Actions, and Implementation.

Today, we would like to hear your opinions and experiences about the topics we discuss regarding the Prepare for Action section of the toolkit. There are no right or wrong answers to any of the questions we will ask. You are the expert on your experience, and your thoughts and opinions are greatly valued and appreciated. We encourage you to speak openly about your opinions and experiences. Anything that you share will be kept private, and your responses will be grouped with answers from other participants. No identifiable information will be known after responses are grouped.

With your permission, I would like to audio record this conversation to ensure that the notes we take are accurate and comprehensive. The recording will be stored securely.

All information collected from these interviews will be housed on a secure drive on the CDC network only accessible to project members and will be shared only in aggregate form. We will not include an names in any reports. The interview should take approximately 30 minutes. If you would like to skip any particular question or would like to stop the interview at any time, please let me know.

**Do you have any questions before we begin? Notetaker documents any questions.**

**Do you agree to participate in this interview?**

⭘ Yes

⭘ No

**Do we have your permission to record this interview?**

⭘ Yes

⭘ No

**[*Start the recorder and state Interviewer name, Date and Participant ID*]**

We would like to confirm that you verbally agree to the consent form and that this conversation will be audio recorded. Again, this information will be housed on a secure drive at CDC and will be shared only in aggregate form. Do you agree to be recorded? _________________

**Pre-Discussion Questionnaire (Cross-sectional survey)**

*Skip this section if this person has filled out the survey prior to the start of this interview and move to the next section.*

*If the person still needs to take the pre-discussion questionnaire, please give that to them now to complete prior to the discussion questions*.

We have provided you with the quantitative questionnaire. Please take the time now to complete this questionnaire before we begin the discussion part of the interview.

**Qualitative Discussion Guide**

Throughout the interview, we are going to ask you questions relating to the implementation of the Environmental Cleaning Toolkit, [INSERT SECTION OF TOOLKIT HERE].

1. “What was your role during this [Insert Section]?”
   1. *Probe: “What did you like about this role? ” “What did you find easy about this role?”*
   2. *Probe: What questions did you have regarding your role prior beginning [Insert Section]? Was this made clear as you carried out [Insert Section]?*
   3. *Probe: “What were some of the difficulties you yourself encountered as you worked through [Insert Section]?”*
2. “What would you say this [Insert Section of Toolkit] accomplished?”
   1. *Probe: “Did this [Insert Section] identify any areas for improvement in environmental cleaning you or the team hadn’t previously considered?”; “What were these improvement areas you hadn’t previously considered?”*
   2. *Probe: “Which tools of [Insert Section] worked well?”;”Can you please elaborate? (If needed)”*
3. “What challenges did your team experience in carrying out [Insert Section]?”
   1. *Probe: “What were the tools that proved particularly challenging?”*
   2. *Probe: “What changes could have been made to make [Insert Section] better or make the steps easier to accomplish?”*
   3. *Probe: How appropriate was the time required to complete [Insert Section]? Can you describe any ways in which it kept you from your regular duties? What about others (leadership, wars staff, cleaning staff, etc).*
   4. *Probe: “How clear or confusing were [Insert missing or incomplete tool abstracted from monitoring checklist]?”*
4. “How has the Toolkit been helpful towards improving environmental cleaning at the facility?”
   1. *Probe: “What changes could have been made to made to [Insert Section] to better meet the needs at the facility?”*
   2. *Probe: “Do the areas highlighted for improvement by this Section match with areas the facility has prioritized recently? What are some examples?”*
5. ***[In reference to any missing items on the monitoring checklist, if applicable]*** “What problems or concerns were there with this part of the [Insert Section]?”
   1. ***Probe: Probing questions will be dependent upon any missing or additional information identified upon review of the associated monitoring checklist from [Insert Section].***
6. “Is there anything else you would like to share, or that you think is important to share regarding the Environmental Cleaning Toolkit that could improve its future use at other facilities?”

That concludes our interview. Thank you again for your time and for sharing your experiences and perspectives while implementing the Environmental Cleaning Toolkit. Your responses will be analyzed together with the responses of others working on the implementation of this toolkit and compiled into a report to improve the use of this toolkit in the future.

**Stop recording.**
