# Supplementary material for: Evaluation of the feasibility, appropriateness, and acceptability of an environmental cleaning program improvement toolkit at a tertiary care hospital in Nigeria
Source: Antimicrob Resist Infect Control. 2025 Apr 18;14:33. doi: 10.1186/s13756-025-01550-5 (PMC12008941; doi:10.1186/s13756-025-01550-5)
Supplement: Supplementary file 3 — Supplementary Material 3 [file 13756_2025_1550_MOESM3_ESM.docx]

**Additional file 3. Cross-sectional survey questionnaire.**

**Environmental Cleaning Toolkit: Cross-sectional Survey Questions**

1. Do you agree to participate in this interview? (If not, please enter “no” below and proceed to the end of the survey without answering any further questions).

- Yes
- No

1. How much do you agree with the following statements in the table below regarding the implementation of [*insert Section A, B or D*] of the Environmental Cleaning Toolkit?

|  | Strongly Disagree | Disagree | Neither agree nor disagree | Agree | Strongly Agree |
| --- | --- | --- | --- | --- | --- |
| The goals of [*insert Section A, B or D*] of the Toolkit were well defined before starting. |  |  |  |  |  |
| The Toolkit is not conducive to team-oriented approaches to problem solving. |  |  |  |  |  |
| My role in this part of the Toolkit was well-defined. |  |  |  |  |  |
| The tools provided were helpful to achieving the section’s goals. |  |  |  |  |  |
| The tools in [*insert Section A, B or D*] were confusing or difficult to follow at times. |  |  |  |  |  |
| This section of the Toolkit demanded too much time away from my regular work duties. |  |  |  |  |  |
| The section’s tools worked well in our facility. |  |  |  |  |  |

1. How would you describe the Toolkit’s influence on your own ability to identify areas for improvement in environmental cleaning at your facility?

- Not influential at all
- Slightly influential
- Somewhat influential
- Very influential

1. To what degree would you consider using this portion of the Toolkit to help make improvements to environmental cleaning at your facility in the future?

- Would not consider using the Toolkit again
- Might or might not consider using the Toolkit again
- Definitely would consider using the Toolkit again

1. Comments (optional):
